# SMARCA4-deficient thoracic sarcoma revealed by metastasis to the small intestine: a diagnostic dilemma

**DOI:** 10.1007/s11748-021-01627-z

**Published:** 2021-04-17

**Authors:** Fatma Khanchel, Raweh Hedhili, Hakim Zenaidi, Imen Helal, Abdelwahed Yahmadi, Hend Ben Néji, Feryel Ksontini, Ehsen Ben Brahim, Raja Jouini, Aschraf Chadli

**Affiliations:** 1 Faculty of Medicine of Tunis, University of Tunis El Manar, 1007 Tunis, Tunisia; 2 Department of Pathology, Habib Thameur Hospital, 1008 Tunis, Tunisia; 3 Department of General Surgery, Trauma and Burns Center, 2074 Ben Arous, Tunisia; 4 Hemophilia Treatment Center, Aziza Othmana Hospital, 1008 Tunis, Tunisia; 5 Medical Oncology Department, Salah Azaeiz Institute, Tunis, Tunisia

## Abstract

SMARCA4-deficient thoracic sarcoma (SMARCA4-DTS) is a recently identified aggressive subtype of sarcoma. We present the case of a 44-year-old man who underwent a surgery for a perforated small intestine. Compued tomography scan revealed a tissular mediastino–pulmonary mass.

Histopathological examination of the intestinal mass shown a malignant tumour with a typical epithelioid and rhabdoid cells, numerous mitoses and large necrosis. A large panel of immunohistochemistry revealed loss of SMARCA4 and SMARCA2 and allowed the diagnosis of SMARCA4-DTS. It is important to consider SMARCA4-deficient thoracic sarcoma in the differential diagnosis of tumours showing suggestive morphologic features in patients of all ages, especially in the case of metastasis associated with thoracic mass.

## Introduction

SMARCA4-deficient thoracic sarcoma (SMARCA4-DTS) is a distinct subset of intrathoracic tumors, recognised for the first time in 2015 by Le Loarer et al. [1]. This tumor is characterized by inactivating mutations of *SMARC4*, a gene encoding the ATPase subunit of the switch/sucrose non-fermenting (SWI/SNF) chromatin remodelling complexes. It has an extremely poor prognosis with metastatic disease at the time of presentation and a median survival of 6–7 months [2]. Recognizing SMARCA4-DTS from other malignant epithelioid tumors is clinically relevant.

We report a case of a SMARCA4-DTS revealed by intestinal metastasis with an emphasis on differential diagnosis.

## Case report

A previously healthy 44-year-old man, with a 25-pack-year smoking history, underwent a surgery for acute peritonitis revealing a perforated small intestine mass with numerous peritoneal nodules and large lymph nodes.

**Fig. 1:**
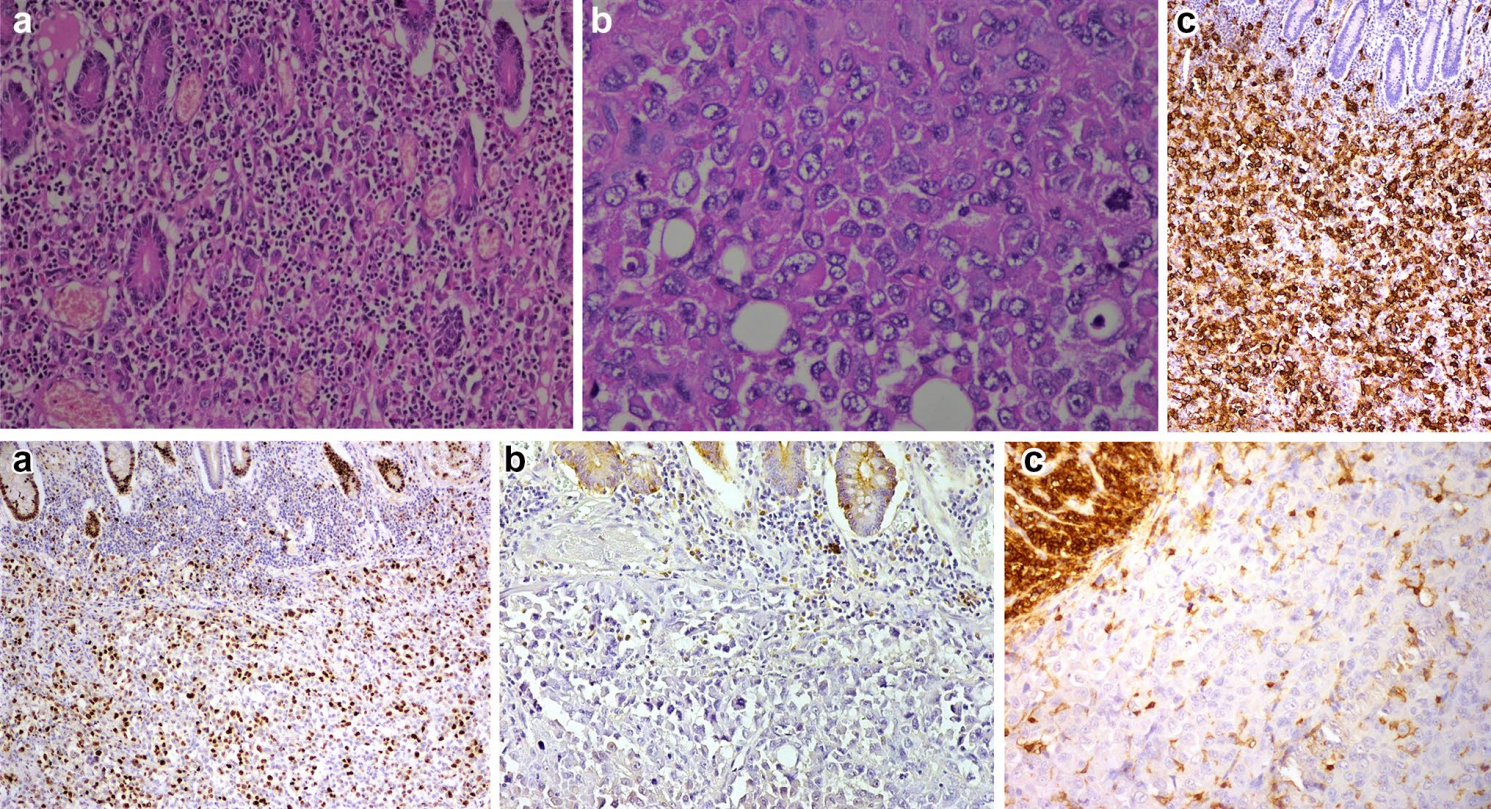
Histopathologic features of intestinal metastase of SMARCA4-deficient thoracic sarcomatoid tumors. **a** H&E Stain (20×) and **b** H&E Stain (40×)—illustrate hallmark morphology: undifferentiated round to plasmacytoid cells with prominent nucleoli, discohesion, and overall monomorphism. Classic rhabdoid cells with hyaline cytoplasmic inclusions indenting the nuclei were present focally in most cases; Immunohistochemistry showed intensive and diffuse expression of vimentine (**c**) and CD34 (**d**). Tumor cells were negative to anti- Pan-CK (**e**) and antiCD45 (**f**)

**Fig. 2:**
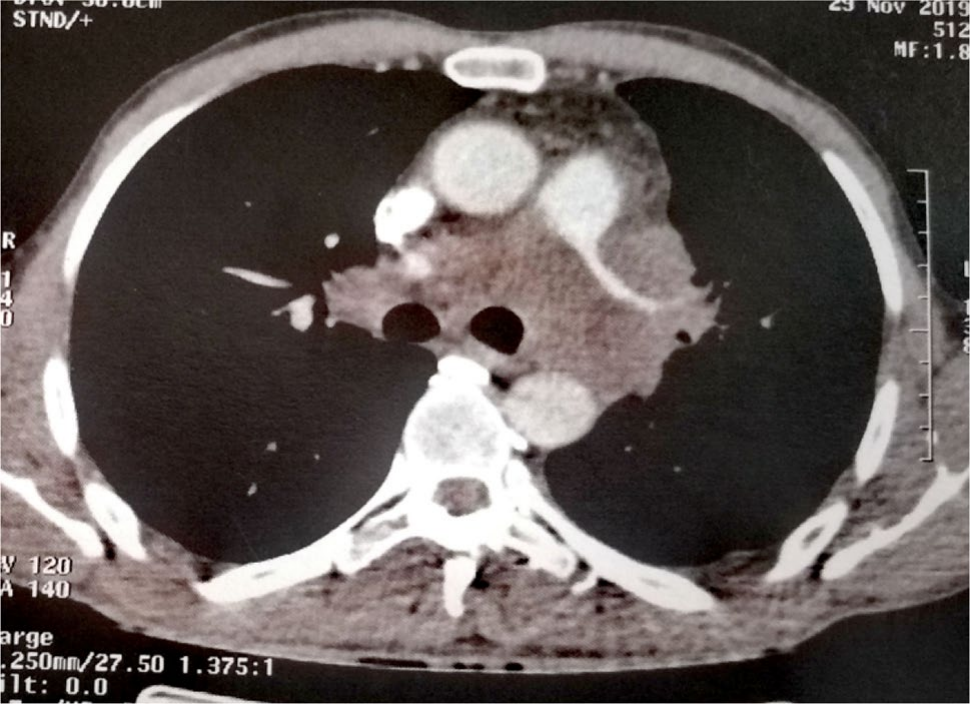
Computed tomography scan of SMARCA4-deficient thoracic sarcomas showed large mediastino–pulmonary mass encompassing left arteries, left common carotid and aortic arch. The mass repressed superior vena cava. This mass was inseparable from latero-tracheal adenomegalies

We received a 26-cm-long segment of the small intestine measuring, two lymph nodes and a peritoneal nodule. The intestine includes an ulcerating and infiltrating tumor of 6 cm. Several samples were taken. Histological examination showed a malignant tumor with poorly cohesive sheets of large cells with an epithelioid and rhabdoid appearance (Fig. 1a). The tumor cells were anysocaryotic without major pleomorphism. Their cytoplasm was globoid and eosinophilic and the irregularly shaped nuclei had vesicular chromatin with large nucleoli (Fig. 1b). Mitoses and areas of geographic necrosis were numerous. Lymph nodes and the peritoneal nodule were massively infiltrated by this tumour. The immunohistochemical study showed a diffuse tumour cell positivity with vimentin, CD34, and SOX2 with more heterogeneous positivity for SALL4 (Fig. 1c, d). It revealed negative expression to epithelial markers CK AE1/AE3, Pan-CK and EMA ruling out the diagnosis of an undifferenciated carcinoma (Fig. 1e). Large cell lymphoma was also excluded by negative expression of lymphoid markers (CD45, CD20, PAX5, CD79a, MUM1, CD138, CD163, CD43, CD3, CD5, CD7, CD68, CD163, TdT, MPO, CD30, CD15, ALK, XCL13, CD21, CD23) (Fig. 1f). Epithelioid sarcoma showing strong and diffuse positivity to EMA and Vimentin was also excluded. In the same way, malignant peripheral nerve sheath tumour and melanoma were ruled out by negative expression of S-100. Also tumour cells did not express neither CD117 nor DOG-1 making the diagnosis of gastro-intestinal stromal tumor unlikely. Negativity to CDK4 and MDM2 eliminated the diagnosis of liposarcoma. Negativity to CD31and Desmin excluded, respectively, a vascular and a muscular differenciation of this tumor. In the same way negativity to synaptophysin, chromogranin and CD56 ruled out a neuroendocrine differenciatin. The proliferation index was estimated at 60% and the PDL1 was < 1%. After this large panel of immunohistochemistry the diagnosis remained doubtful. A computed tomography scan, performed after the intervention, revealed a tissular mediastino–pulmonary mass measuring 47 × 100 × 116 mm encompassing left pulmonary arteries, left common carotid and aortic arch and repressing superior vena cava (Fig. 2). This mass was inseparable from latero-tracheal enlaged lymph nodes. In the abdomen, there was an 80 × 70 × 64 mm tumour residue, large lymph nodes, and bilateral adrenal masses.

**Fig. 3:**
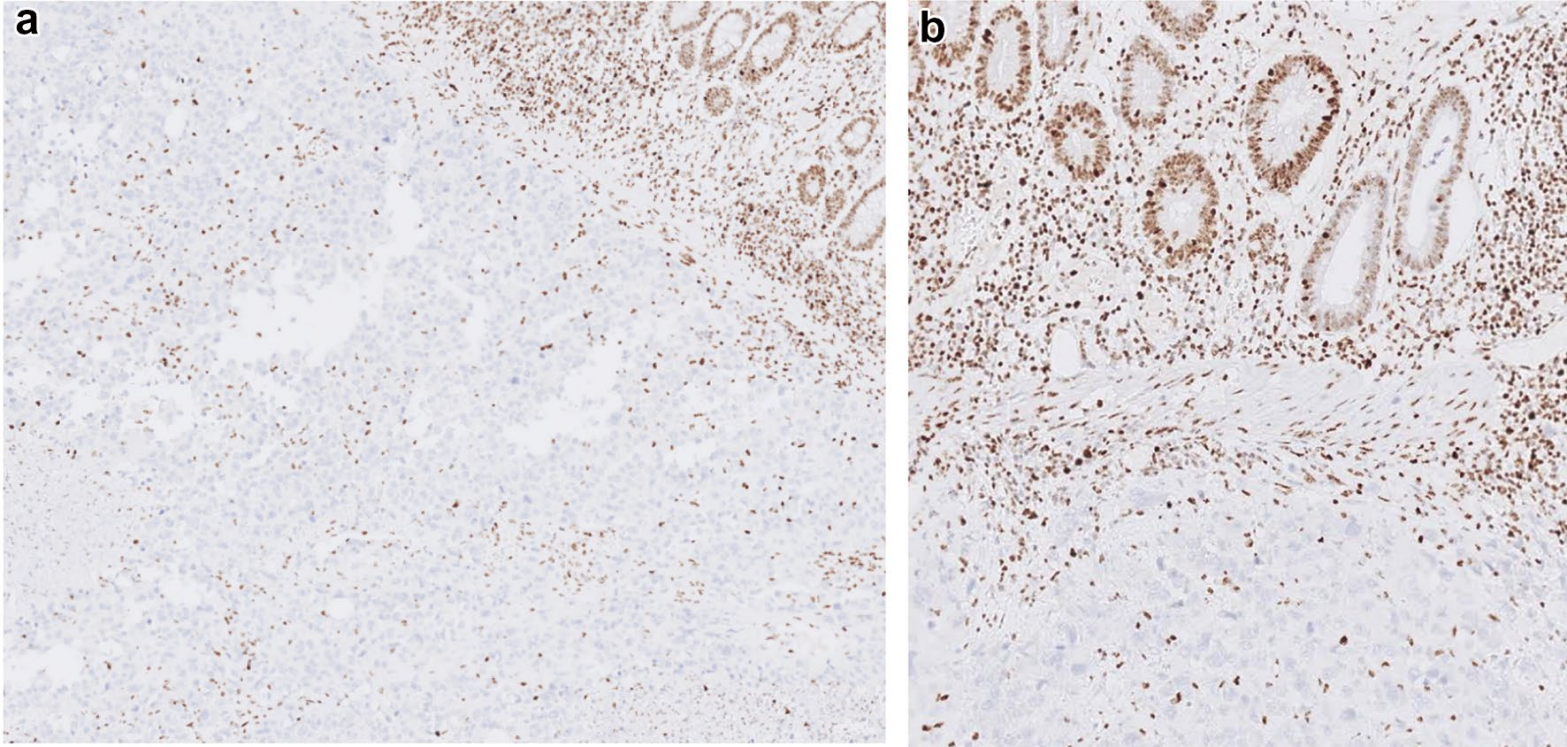
SMARCA4-deficient thoracic sarcomas demonstrated dual complete lack of expression of SMARCA4 in (**a**) and SMARC A2 in (**b**). Note endothelial and inflammatory cells as internal positive controls (10×)

Based on computed tomography scan data a complementary immunohistochemical investigations were performed and they revealed that tumor cells exhibited a loss of expression of both SMARCA4 and SMARCA2 while SMARCB1 was retained (Fig. 3a, b).

Thus morphology and immunophenotype, coupled with the computed tomography data and clinical history, were consistent with the diagnosis of SMARCA4-deficient thoracic sarcoma to the small bowel.

The patient received two cycles of chemotherapy with gemcitabine and cisplatin. He died 6 months after the diagnosis from a digestive haemorrhage.

## Discussion

SMARCA4-DTS is first described in 2015 by Le Loarer et al. [1]. The authors compared transcriptomic profiles of unclassified thoracic sarcomas showing SMARCA4 inactivation with those of SMARCA4-mutated small-cell carcinomas of the ovary, hypercalcemic type (SCCOHTs), SMARCB1-inactivated malignant rhabdoid tumors (MRTs), and lung carcinomas (of which 5–10% has SMARCA4 mutations) [1]. As a result, SMARCA4-DTSs were found to be genetically different from lung carcinomas while they displayed a closer molecular relationship to SCCOHT and MRTs [1].

The *SMARCA4* gene, located on chromosome 19p13, encodes the BRG1 protein. The *SMARCA2* gene, located on chromosome 9p24 encodes the BRM protein. These two proteins are mutually exclusive catalytic subunits of the SWI/SNF chromatin-remodelling complex which is formed by multiple other proteins, among which INI-1 (encoded by *SMARCB1* gene). By regulating transcription, promoting cell differentiation, and repairing the DNA, the complex acts as a tumour suppressor [3]. Studies have demonstrated that up to 20% of human malignancies contain a mutation of one of the subunits of the SWI/SNF complex and span many tumors types [4].

Usually, clinical presentation of SMARCA4-DTS is a compressive and infiltrative large mediastinal mass, rarely found in the lung [2]. They occur in middle-aged males with a heavy smoking history [1]. Most of the cases present with metastatic disease involving lymph node, bone, adrenal glands, skin [2, 5–7]. In addition, these tumours have a predilection for bulky peritoneal metastases, which may raise a consideration for an abdominal primary like in the case of our patient [2]. Hence, to have a correct diagnosis, thorax radiological features are very helpful.

In typical cases, SMARCA4-DTSs is a poorly differentiated neoplasm of round to epithelioid cells with prominent cytological atypia organized in a solid pattern and showing rhabdoïd differentiation [1]. Mitosis and necrosis are common [1]. Immunohistochemical analysis of SMARCA4 and SMARCA2 expressions, respectively based on BRG1 and BRM immunohistochemistry, shows a dual loss [1]. Expression of SOX2 is often diffuse and in most cases epithelial markers (CK AE1-AE3 or EMA), CD34, and/or SALL4 are focally expressed [1]. Other markers are generally requested to rule out differential diagnoses. In our case, undifferenciated carcinoma is a differential diagnosis but it is immunopositive for epithelial markers CK AE1/AE3, Pan-CK and EMA. Large cell lymphoma is also a differential diagnosis but was ruled out by negative expression of lymphoid markers. Epithelioid sarcoma shows a strong and diffuse positivity to EMA and vimentin. Malignant peripheral nerve sheath tumour and melanoma were also ruled out by negative S-100 immunohistochemistry. The dual loss of SMARCA4 and SMARCA2, the diffuse positivity for CD34 and SOX2, the heterogeneous positivity for SALL4, in addition to thorax radiological features confirmed the diagnosis of a SMARCA4-DTS.

In 2019, Perret and Le Loarer’s group in France proposed three criteria, deemed sufficiently significant in terms of specificity and sensitivity, based on which the diagnosis of SMARCA4-DTS intrathoracic malignancies ought to be carried: (1) rhabdoid or poorly differentiated phenotype, (2) complete loss of expression of SMARCA4 and SMARCA2, and (3) focal or diffuse expression of at least two of the following markers: SOX2, CD34, or SALL4 [8]. But the diagnosis is particularly difficult in the case of inaugural metastases, which is similar to our case. The main differential diagnosis of SMARCA4-DT in the intestinal tract are epitheloid gastro-intestinal stromal tumor, melanoma, undifferentiated carcinoma, and epithelioid malignant nerve sheath tumor. Even though the immunophenotype of SMARCA4-DTS is quite specific, appropriate clinicopathologic correlation should be performed in all cases to avoid misclassification [8].

The prognosis for SMARCA4-DTS cases is poor because treatment strategies for these tumors have not been established. The estimated median overall survival duration is 7 months [2]. Presently, specific therapies combining the enhancer of zeste homolog (EZH2) inhibitors, used in patients with abnormalities in the SWI/SNF complex protein, and the topoisomerase II inhibitor are widely supported to treat SMARCA4-deficient malignancies [9]. A study published by Takeda K et al. suggests that anti-PD-1 blockade may be effective for SMARCA4-DTC expressing PD-L1 [10]. These results remain to be validated.
